# An In Vivo Study of the Effects on Serum Glucose, Amylase and Histopathology of the Feline Pancreatic Tissue Treated by Focused Ultrasound

**DOI:** 10.1371/journal.pone.0088815

**Published:** 2014-02-18

**Authors:** Ying Mao, Liaoqiong Fang, Liang Ai, Chongyan Li, Zhibiao Wang, Junru Wu, Jin Bai, Faqi Li

**Affiliations:** 1 State Key Laboratory of Ultrasound Engineering in Medicine Co-founded by Chongqing and the Ministry of Science and Technology, College of Biomedical Engineering, Chongqing Medical University, Chongqing, China; 2 National Engineering Research Center of Ultrasound Medicine, Chongqing, China; 3 Department of Physics, University of Vermont, Burlington, Vermont, United States of America; The Ohio State University, United States of America

## Abstract

Pancreatic cancer is one of the most malignant neoplasms originating in the digestive system. Focused ultrasound (FUS) treatment instead of the surgery operation has been used to treat Pancreatic cancer noninvasively in clinical trials. The endocrine and exocrine glands in pancreas provide the two unique functions for a person to be healthy. It is critically important to find out if the FUS treatment can still keep the normal functions of the two glands. The goal of this study is to examine and analyze changes in histopathology and serum glucose and amylase levels of the targeted *in-vivo* felines after the FUS treatment. Various percentage volumes of pancreas of felines were insonified. The FUS treatment (7.5 MHz of central frequency; 5 W of acoustical power; transducer f-number  = 0.33; 6 s insonification time per point) effectively generated coagulative necrosis at the insonified site while leaving tissue outside the insonified site intact. It was also observed that all felines endured well with the FUS treatment; changes introduced to pancreatic tissue after up to 50% of a pancreas by volume was insonified by the FUS procedure did not affect its normal endocrine and exocrine functions.

## Introduction

Pancreatic cancer is one of the most malignant neoplasms originating in the digestive system. It ranks the 13th in terms of morbidity and the 8th among tumor mortality [1]. This kind of malignant cancers has poor prognosis with early invasion and metastasis. The 5-year survival rate is merely 3–5%, and it may reach 15% if it is early detected [2]–[4]. In the past few decades, pancreatic cancer is mainly treated with surgery, radiotherapy, and chemotherapy. It is found that any single and the combination of existing treatments other than surgery are not effective [5]. Surgery, although it is invasive and its resection is low, remains the sole “arsenal” against the pancreatic cancer. About 85–90% of patients, when are diagnosed to suffer cancer, might have already missed the best time for surgery due to the local invasion and metastasis [3], [6], [7]. Pancreatic neoplasm is known to be less sensitive to chemotherapy; the success rate with taking gemcitabine, one of the most effective tumor “killer”, stands only at 23.8% [8]. Radiotherapy has its restriction since surrounding tissues including liver, kidney, gastrointestinal tract and bone marrow are vulnerable to radiation. Thus as a tradeoff radiotherapy dosage is usually controlled [9]. Therefore a new localized, noninvasive and effective modality is urgently needed. FUS is recently found to be an emerging non-invasive technique; it meets the challenge of the urgent need of pancreatic cancer treatment. The advantages of FUS compared with the existing other therapeutic modalities of cancer treatment are: (1) Its good tissue penetration ability; (2) relative ease with focalization on the targeted tissue. The physical mechanisms of FUS include mechanical effects due to acoustic cavitation (violent bubble-activities) in situ and thermal effects generated by ultrasound absorption by the targeted cancer tissue and the local temperature could reach over 65°C almost instantaneously [10]. The above combined effects may induce protein degeneration and coagulative necrosis, while leaving surrounding tissues intact [11]. FUS has now been recognized to have excellent therapeutic effects in treating hysteromyoma, breast cancer, hepatoma, pancreatic cancer, bone tumor and nephroma [12]. Compared with other thermotherapies, FUS excels in its non-invasiveness, adaptability, real-time monitoring and the ability to deal with a large tumor and a tumor next to major vessels [11]. Several clinical trials were taken or are taking place to treat patients with progressive pancreatic cancer using FUS alone or combined with gemcitabine. The early results of those trials have exhibited that FUS has good therapeutic and pain control effects (overwhelming pancreatic cancer patients suffer serious pain). Very few side-effects well tolerated by patients were found in those trials[13]–[16], for example, hyperamylasemia, hyperlipsemia with mild abdominal pain occurred for few patients, but their elevated pancreatic enzyme levels (amylase and lipase) decreased within 7days [16].

Pancreas stands out among all organs with its unique function of endocrine and exocrine. Therefore, before the FUS procedure is considered for treatment, the issue how pancreatic tissue responds to the FUS exposure should be systematically investigated. Endocrine plays an essential role in regulating metabolism of glucose, fat and protein. Maintaining normal serum glucose after the FUS treatment is essential for the FUS to be accepted as a potential alternative modality for treating the pancreatic cancer. Further, the pancreas enzymes could digest the pancreas tissue itself under certain situations, resulting in severe complication such as acute necrotizing pancreatitis or pancreatogenous peritonitis. So safety is the most important issue to be considered in the therapy of pancreatic disease. Although the chronic pancreatitis literature suggested that a substantial proportion of the pancreas must be affected in order to develop diabetes due to pancreatic endocrine dysfunction and that clinically significant protein and fat deficiencies do not occur until over 90 percent of pancreatic function is lost [17], a systematic study to find out how FUS treatment would influence exocrine function is still warranted. Up to our knowledge, there are only two studies done by Hwang (2009) and Xie(2011) which demonstrated the applicability and safety of FUS through an experiment on normal swine pancreatic tissue [18], [19]; however they did not investigate the relationship between percentage volumes of pancreas necrosis produced by FUS treatment and dysendocrinism and exocrine disorders.

It is our intention to investigate the possible changes of endocrine, exocrine and histopathology caused by FUS treatment on felines' pancreatic tissue under an *in vivo* condition. The study, we believe, is important and necessary to assure the effectiveness and safety for FUS treatment of the pancreatic cancer.

## Materials and Methods

### Laboratory animals

The experimental and animal care protocols were approved by the Chongqing Medical University review board in compliance with the guidance for the care and use of laboratory animals from the Ministry of Science and Technology of the People's Republic of China and in reference of the American Medical Association Family Medical Guide, Random House, P. 499, 1987). Fifty four healthy felines (cats) with sex ratio of 1∶1 of 1 to 2 years age and weighed between 3.0 to 4.0 kg were obtained from the Animal Center of the Chongqing Medical University. They were randomly divided into 3 groups: 1, the USS group (22 cats, an half of one lobe or about 25% of whole pancreas was insonicated since two lobes have roughly equal volume); 2, the USL group (22 cats, insonication volume being one whole lobe about 50% of whole pancreas) and 3, the control group (10 cats, sham exposure). They were all deprived of the solid food for one day (ambrosia) and water for 12 hours before the procedure. In the following 3 days after the treatment, all cats were fed a standard liquid diet and received intravenously ranitidine (50 mg) in 5% glucose saline 150 ml plus 10% glucose solution 150 ml daily. The amount of 600 hundred units of penicillin was administered intramuscularly to each cat once a day for 3 days continuously after operation. Ten cats' blood was collected randomly for serum glucose and amylase tests shortly after operation and on the 3rd, 7th, 14th, 21th days after the FUS procedure. Three cats each time were randomly chosen immediately after and on the 3rd, 7th, 14th, 21th days after the procedure in insonication groups to be sacrificed for histopathological tests. The rest of 7 cats received euthanasia at the end of experiments according to the protocol.

### Focused ultrasound system

**Figure 1:**
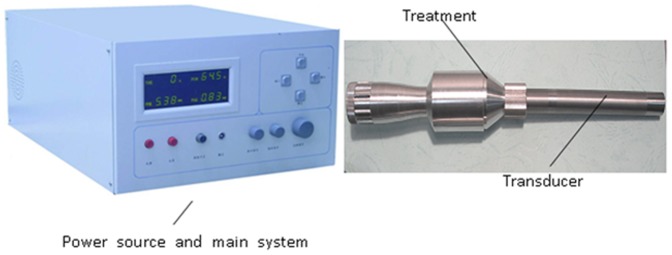
Focused ultrasound treatment system.

**Figure 2:**
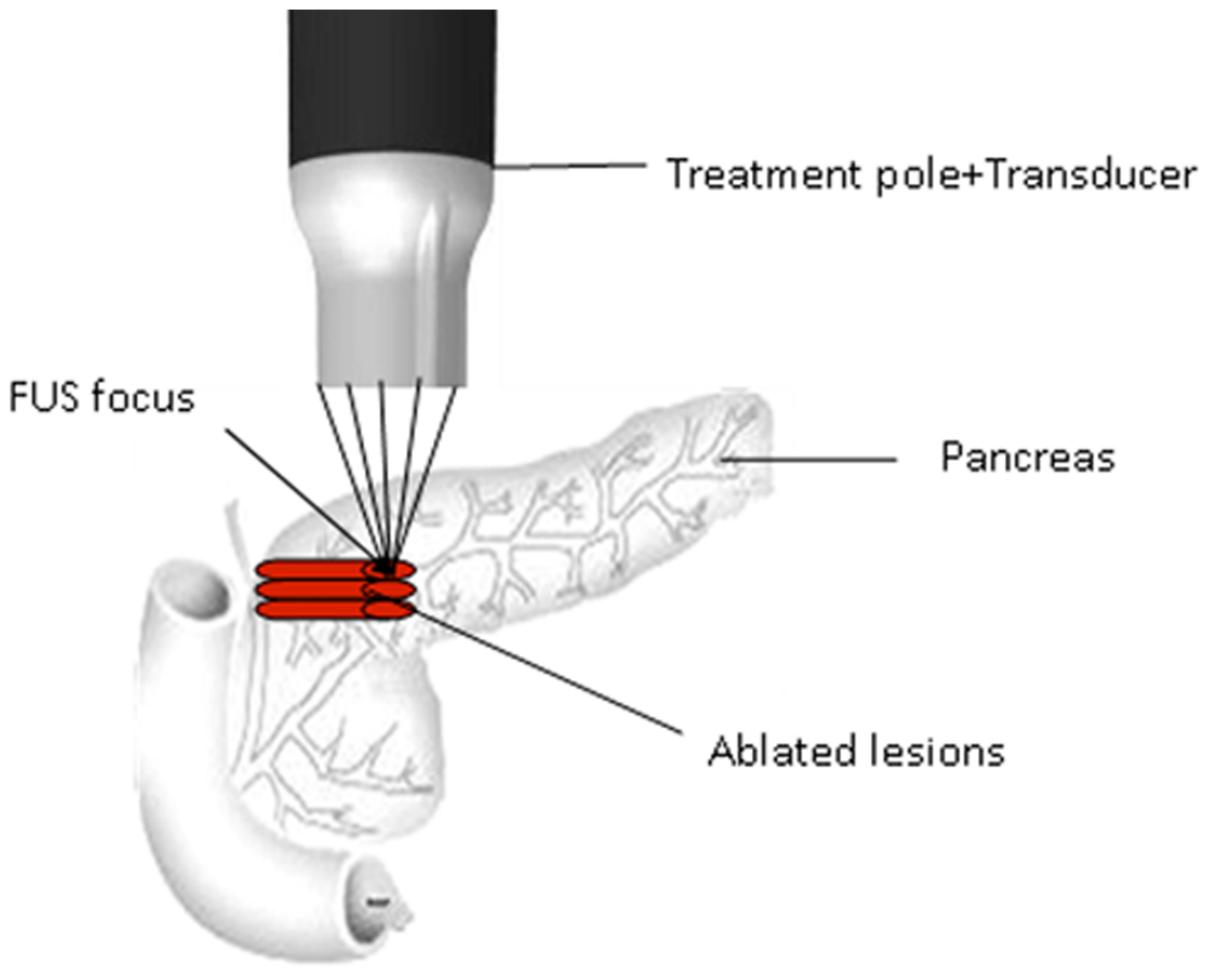
Illustration of FUS treatment on pancreas.

The focused ultrasound therapeutic apparatus used in this study was designed by Chongqing Haifu (HIFU) Medical Technology Co. Ltd (Chongqing, China) which consists of the main system, a power source, a treatment pole, a transducer, and a degassing and dehydration water system which removes gas from water to less than 3 ppm and pumps water to the applicator (Fig. 1). The main system and the power source supply the electrical voltage to the treatment pole which boosts the voltage applying to an ultrasound transducer. The ultrasound beam is generated by an focused ultrasound transducer. The central frequency of the focused ultrasound transducer is 7.5 MHz, its aperture is 12 mm, and the focal length is 4 mm. The acoustic field has been reported in earlier paper by Zhang Q et al. (2011) [20].The acoustic power output is measured to be 5 W by the radiation force measurement technique [10]. During the treatment, an operator usually holds the treatment transducer against the targeted area directly using saline as the coupling medium (Fig. 2).

### The FUS treatment

Felines were depilated with 8% sodium sulfide solution and narcotized with ketamine (10 mg/kg) intramuscularly. Skin was routinely disinfected and covered by a piece of sterile cloth specifically made for the purpose of surgery. The portion of the body directly above the pancreas was cut to expose stomach and duodenum. Pancreas is about 10 cm in length, 1–2 cm in width, 3–5 mm in thickness, consisting of two roughly identical lodes, namely the duodenal lobe and the greater gastric curvature lobe, was pulled outside the body. A disinfected treatment transducer was placed in direct contact with the surface of pancreas for insonification to ultrasound (7.5 MHz of central frequency, 5 W of acoustical power or 2500 W/cm^2^ of acoustical intensity). Insonification was done by moving the transducer from points to lines, then to planes. It stayed at each point for 6 s until visible coagulation necrosis appeared, and then was moved to the next point as shown in Fig. 2. After 6 s exposure, the local temperature at site raised up to 60°C. The feline pancreas is only 3–5 mm in thickness, and the focal length of the transducer is 4 mm, therefore, only one plane of tissue could be treated. For the USL group, insonification was done on 50% of whole pancreas; while for the USS group, only 25% volume of pancreas received treatment. The volumes were estimated by using a digital vernier caliper. During ultrasound exposure, saline was constantly applied on tissue surface both for moisturizing and the acoustic energy coupling. Immediately after the FUS treatment, intactness of surrounding tissue was examined and measured. Then, the pancreas was gently put back to the original position, then muscle and skin were properly sutured.

The average insonification period of the USS group was 60 s whereas for the USL group was 120 s. The control group was similarly treated except that the electrical voltage to the FUS system was turned off.

### Signs and symptoms

Post-operative signs and symptoms which include mental condition, feeding, body temperature, complication and adverse effects (Celiac infection, fever, peritonitis, and pancreatic leakage etc.) were monitored using the routine equipment.

#### Serological tests

The amount of 3 ml of blood was obtained from the femoral vein for serum tests before and immediately after FUS treatment and on the 3rd, 7th, 14th days after the treatment (10 cats randomly chosen for each data point). Serum level of glucose was measured with glucose oxidase-peroxidase method and that of amylase was measured using the enzyme kinetic method as described previously [21], [22].

### Histopathology

Insonicated pancreatic tissue samples by the FUS were taken shortly after the FUS procedure and on 3rd, 7th, 14th and 21th days after the operation, and fixed with 10% methanal, sliced, dyed in HE and examined under an optical microscope for pathological and structural comparisons of the insonicated site and its surrounding tissue. Tissue obtained was also preserved under 4°C and 2.5% glutaraldehyde, rinsed, pre-fixed, rinsed, fixed, dehydrated, embedded and cut into slices. These slices were then dyed in uranyl acetate and citrate acid lead, and examined under an H-600 transmission electron microscope (Hitachi, Japan) for pathological and structural changes.

### Data analysis

Due to the nature of the data, nonparametric statistics were carried out in all instances. Differences between medians of treated and control groups were compared using the Wilcoxon rank sum test for paired data. Statistical significance was defined as P<0.05.

## Results

### Changes due to FUS treatment

Mild fever was observed among all the cats after operation, with rectal temperature of 39.0–40.1°C (feline normal temperature: 38.7±0.5°C), which lasted for 1 to 2 days before subsided on their own accord. All of them experienced restlessness and poor appetite after 3 days of the operation. One of the USL group died on 14th day of choleperitonitis due to bile spillage revealed by autopsy. A tentative decrease in their body weight of all cats was observed; but difference in control group and FUS groups were found insignificant. The body weight recovered 7 days after the treatment.

### General appearance

Pancreas, normally pink and soft, turned whitish yellow and hard with higher temperature immediately after insonification, and surrounding tissue was in congestion and edema after several seconds. On the 3rd day after operation, obvious hyperemia and edema zone existed around irradiation site and there was a clear boundary between hyperemia and normal zone. On 7th day, the exposure site was still whitish yellow and hard, the congestive zone vanished, abdomen saw obvious adhesion. On 14th day, adhesion of abdominal cavity was mild, and irradiation site exhibited almost normal pink color but was still hard in texture. On 21st day, abdominal cavity was free from adhesion, and irradiation site turned normal pink, with hard and granular texture.

### Changes of histology of the coagulative necrotic area of the pancreas

**Figure 3:**
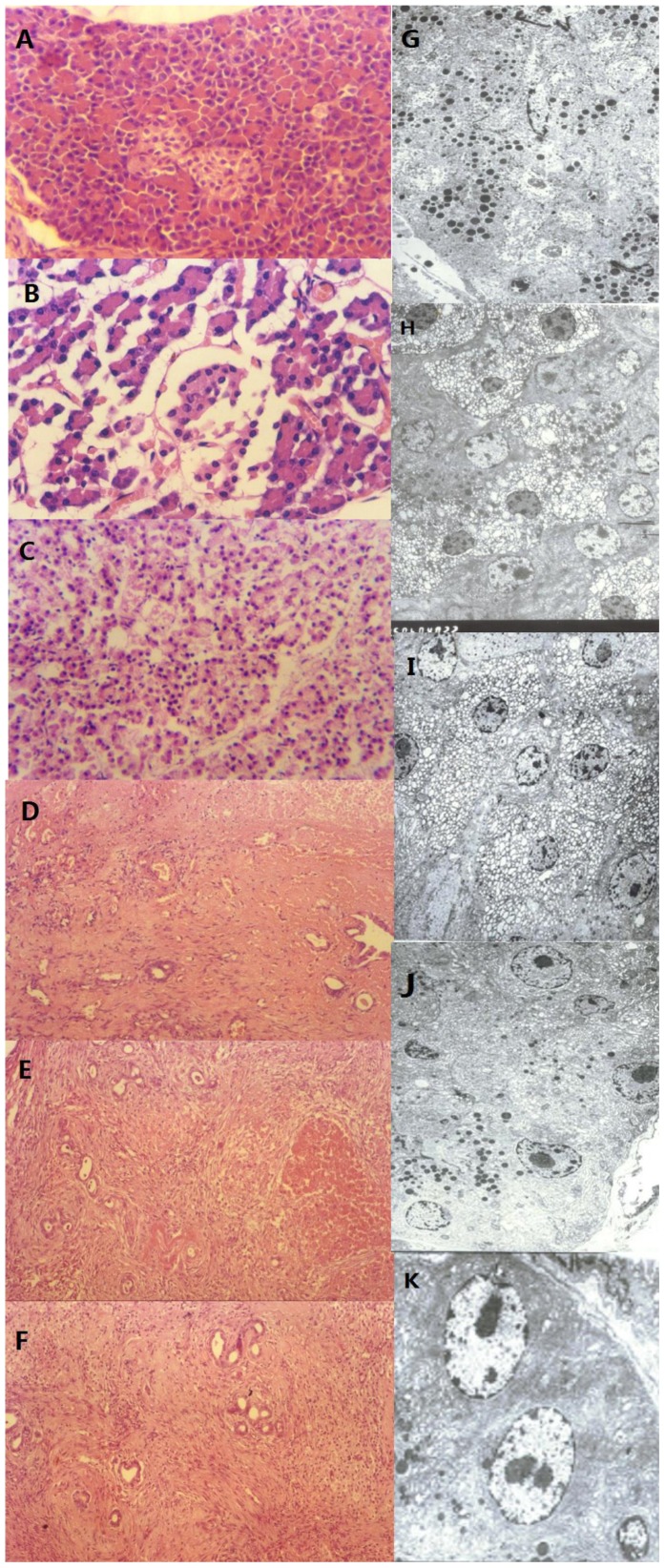
Optical and electron microscope performance of insonified and non-insonified sites after Focused Ultrasound: A (H.E×200), Immediately after FUS, the noninsonified site, islets and exocrine cells were normal in morphology and structure; B(H.E×400) and G(×4000), immediately after Focused Ultrasound, cells in the insonified sites started irreversible necrosis process;C (H.E×200) and H(×5000), 3 days after treatment, the acinar structures were “destroyed,” karyolysis, nuclear fragmentation were observed. D (H.E×100) and I (×3500), 7 days after operation, repair process started and newborn pancreatic ducts formed; E (H.E×100) and J(×4000), 14days after operation, insonified site was replaced by fibrous connective tissue and abundant newborn pancreatic ducts formed; F(H.E×100) and K(×4000), 21days after operation, the necrosis site was replaced by fibrous tissue, pancreatic duct hyperplasia was clearly observed.

Using an optical microscope immediately after FUS, cells of insonified site was found to suffer pyknosis, with smaller cellular size, decreased cytoplasm, condensed nucleus and marginalization of chromatin immediately after FUS treatment; but cellular outline was complete, cell shape was recognizable and structure was loose (Fig. 3B). In non-insonified site, islets and exocrine cells were normal in morphology and structure (Fig. 3A). On the 3rd day, the acinar structure was destroyed at the insonified area, karyolysis,nuclear fragmentation and large of neutrophils infiltration were observed clearly (Fig.3C). On the 7th day, outline of pancreatic acinus was discernible at insonified site, necrosis, cytolysis, vestige of nucleus, distinct proliferation of granulation tissue and formation of new pancreatic duct were observable (Fig. 3D). Two weeks after the procedure, tissue of insonified site was replaced by fibrous connective tissue; more new pancreatic ducts were seen, whose structure was generally normal (Fig. 3E). On the 21st day, the necrosis site was replaced by fibrous tissue, pancreatic duct hyperplasia was clearly observed, whose morphological structure was normal (Fig. 3F).

Under an electron microscope, shortly after FUS, exocrine cells in pancreas were seen with distinct outline, edema, karyolysis, damaged nuclear membrane and granular necrosis of parts of cytoplasm (Fig. 3G). On the 3rd day, cell necrosis appeared, membranous structure in cytoplasm was intumescent, and vacuolation was seen in mitochondria, part of cells turned mesh-like (Fig. 3H). On the 7th day, necrosis was severe, with petechial hemorrhage and indistinct outline; most cells turned mesh-like; karyopycnosis and plasmolysis were observable in some cells; eosinophile granulocyte and plasmocyte were found to infiltrate in the boundary area, and there collagenous fibers and fibrocyte existed (Fig. 3I). On the 14th day, most cells turned mesh-like, petechial hemorrhage was observed, inflammatory cell infiltrated, and proliferation of fibrocyte was prominent in some area (Fig. 3J). On the 21st day, nuclei of exocrine cells were mostly normal with muchendoplasmic reticulum and some swollen chondriosome (Fig. 3K).

### Change of serum glucose level

**Figure 4:**
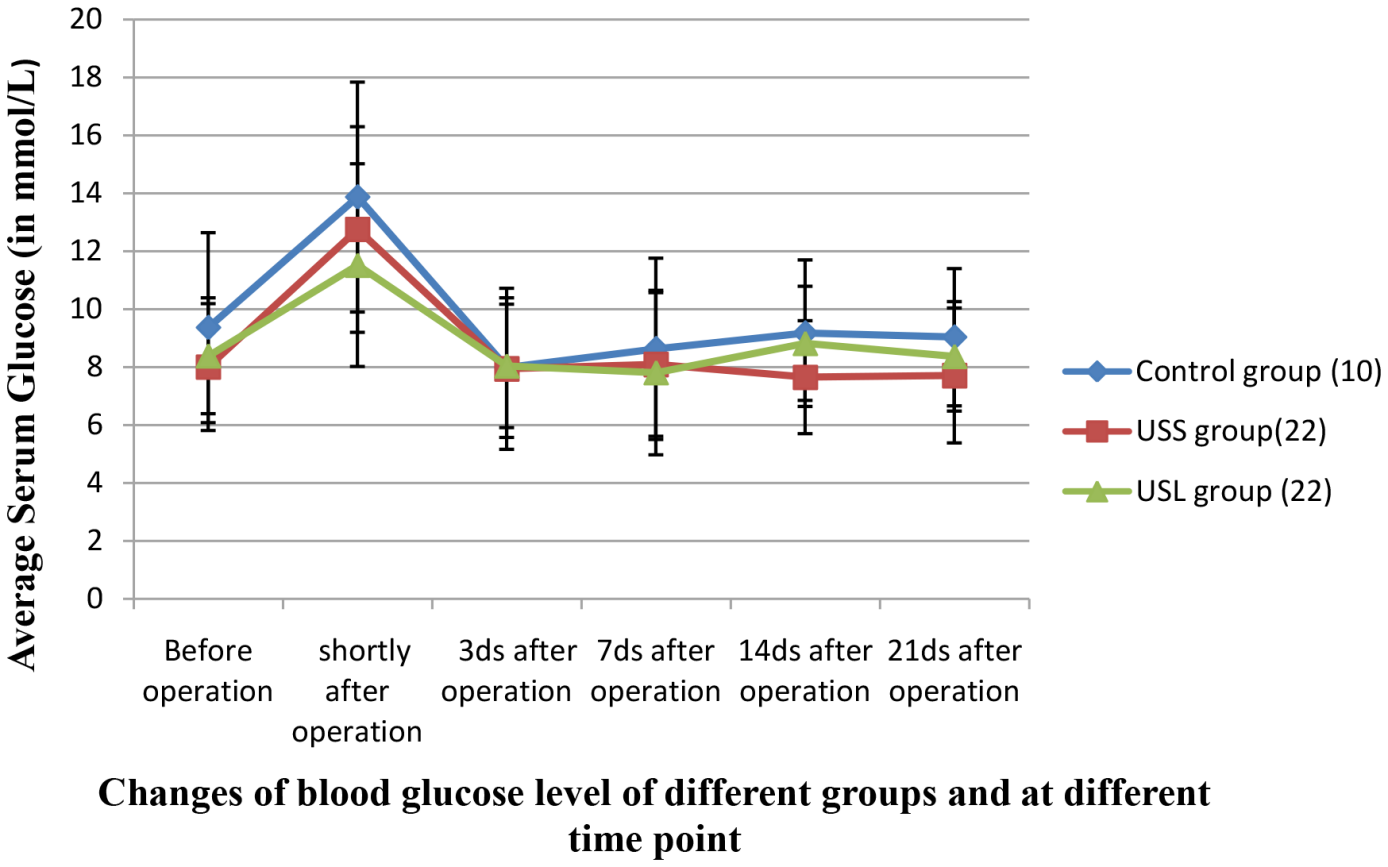
Changes of serum glucose level of different groups at different time-scales.

Serum glucose peaked at shortly after operation (P<0.05) and returned to normal on the 3rd day after the procedure (P>0.05), without distinct differences among groups (P>0.05); such change was consistent among the USS, USL and control groups as shown in Fig. 4.

### Change in serum amylase level

**Figure 5:**
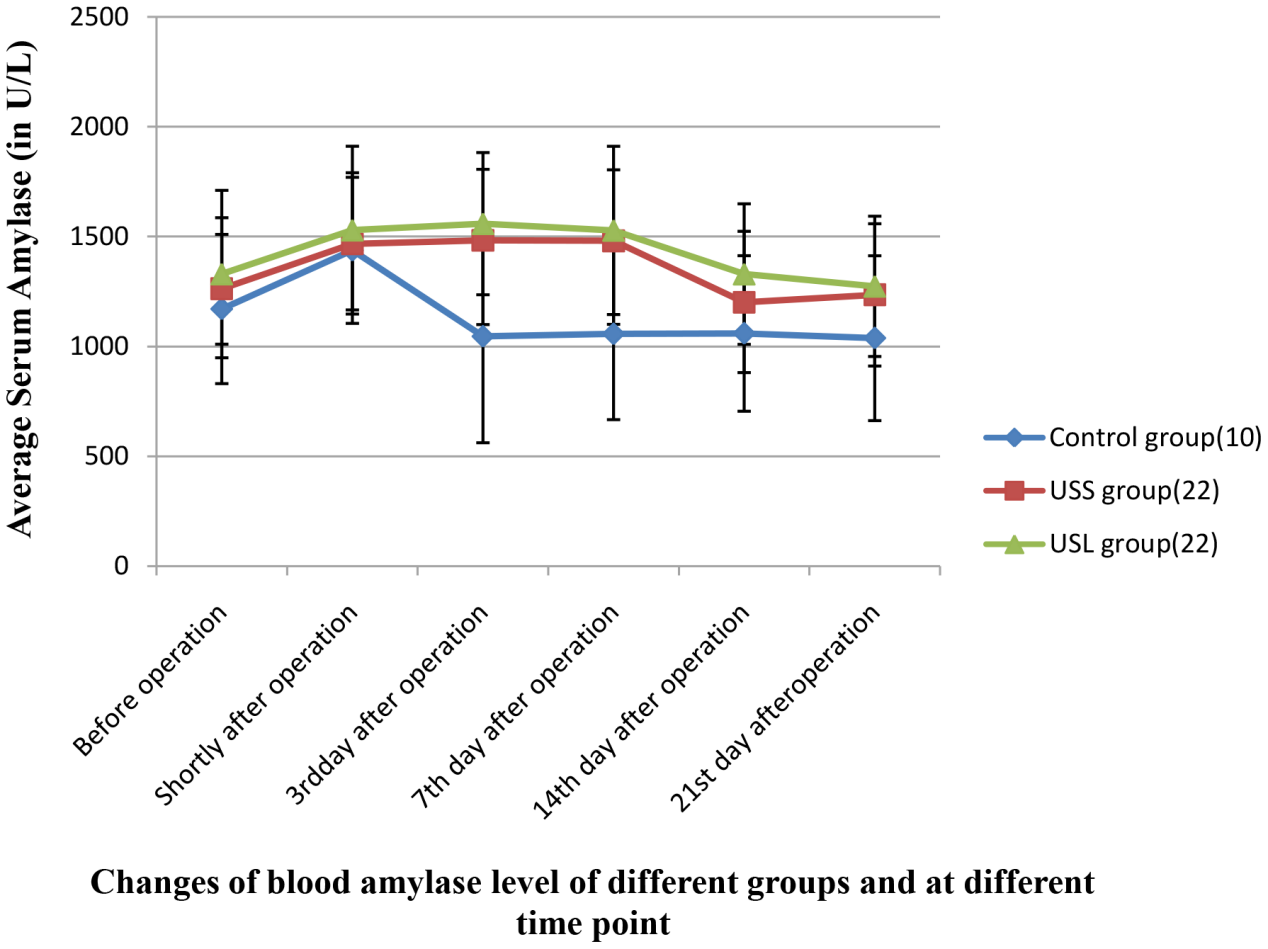
Changes of serum amylase of different groups at different time-scales.

Change in serum amylase level was found to be in a similar fashion between USS and USL groups (Fig. 5): amylase level rose immediately after operation and continued to rise on 7th day (P<0.05), and decrease to normal on the 14th day and remained thereafter (P>0.05). For the control group, it was also elevated immediately after the procedure, but fell to normal at the end of the 3rd day and remained thereafter. Quantitative differences between USS and USL groups were not significant (P>0.05), and those between FUS groups and the control group were significant on the 3rd and 7th day (P<0.05).

## Discussion

Safety concern of cancer treatment by FUS is always an essential issue before it can be accepted by the medical community. “How pancreatic tissue responds to FUS exposure?” was the question that doctors concern mostly. Our study has shown that for the case up to 50% volume of pancreatic tissue of felines was insonicated by FUS the serum glucose of the felines was maintained at the similar level with those of the control group, indicating that excretion of insulin may remain largely intact even up-to-50% pancreatic tissue suffered necrosis. Furthermore, it was manifested by histopathology that FUS “destroyed” pancreatic tissue at insonified site leaving that of non-exposed tissue unharmed. The rising of serum glucose immediately after insonification is believed to be due to the stress not directly related to the FUS treatment. The serum glucose of the insonicated groups changed in a similar manner as that of the control group. From these findings, we may conclude that islet cells outside the FUS treated site were intact in structure and function. FUS has minimal impact on excretion of glucose regulating hormone and therefore post-operative insulin replacement therapy become unnecessary.

Serum amylase level had a hike in 24 h after the onset of acute pancreatitis and returned to normal after 5 to 7 days and maintained that level thereafter. It is considered to be one of the key indicators of pancreatitis for both sensitivity and specificity [23]–[25]. In this study, change of pancreatic exocrine, monitored through amylase level, was consistent with change of observed histology. The rising of amylase shortly after the procedure is due to the destruction of pancreatic acinar cells, released from the destruction of pancreatic cells. On the 3rd day after the procedure, it was revealed by the histology examination that hyperemia and edema zone existed around the insofication sites and serum amylase elevated. The rise of amylase may result from secretion of normal pancreatic cells, which penetrated through destroyed intercellular substance before joining serum stream of vessels. The serum amylase level peaked on the 7th day, then it edged down in the FUS groups two weeks later, this is consistent with the change of histology. New abundant pancreatic ducts were seen after 2 weeks, the structure was generally normal. On the other hand, the differences of amylase level between FUS groups and control group were significant 3 days after surgery (P<0.05) and were maintained until 7th day after treatment, indicating that the rise of amylase clearly associated with FUS induced pancreatic injury but the reversible rise does not harm the individual function. Histopathological study also revealed that new pancreatic ducts formed on the 7th day and fibrous tissue completely substituted destroyed one on the 21st day; the fibrous tissue became home to abundant newborn pancreatic ducts with normal shape. With the increase of pancreatic ducts, serum amylase level decreased back to a level before treatment, which signaled that pancreatic ducts “damaged” by FUS could be repaired gradually and resume the function of secretion. FUS caused necrotic pancreatic tissue which was first surrounded by inflammation and isolated by fibrous connective tissue, then was replaced by fibrous scar. New and normal pancreatic ducts gradually formed in the fibrous tissue. In this study, bile spillage was found for one feline but its bileduct was intact, so the bile was suspected to leak from the junction of pancreaticobiliary duct. It is speculated that the ultrasound treatment transducer was kept too close to the pancreas, which locates next to duodenum, to hurt that duodenum wall. It should definitely be avoided in clinical practice.

## Conclusions

In summary, this experiment demonstrated that FUS treatment effectively generated necrosis on pancreatic tissue at the insonicated site while leaving those outside the site unharmed; the FUS procedure had minimal influence on endocrine and exocrine of pancreas and did not cause complications such as severe acute pancreatitis; the “destroyed” pancreatic tissue was replaced by fibrous tissue; the treated pancreatic ducts were renewable for the cases that up to 50% volume of a pancreas was insonicated by FUS. In future, we will continue expand our research to increase the insonicated volume of a pancreas by the FUS procedure beyond 50% by volume.
